# Ruptured Duodenal Varices Successfully Managed by Endoscopic N-butyl-2-cyanoacrylate Injection

**DOI:** 10.4021/jocmr943w

**Published:** 2012-09-12

**Authors:** Hyung Hun Kim, Sung Eun Kim

**Affiliations:** a Department of Internal Medicine, Kosin University College of Medicine, Busan, Korea

**Keywords:** Duodenum, Varice, Rupture

## Abstract

Bleeding from ectopic varices is rare and accounts for only 1% and 5% of all variceal bleeding. However, once the bleeding starts, it becomes difficult to control and is sometimes fatal. We faced a 65-year-old man with ruptured duodenal varices and injected N-butyl-2-cyanoacrylate into the spurting duodenal varices. As a result, oozing was successfully controlled. Subsequently, the patient remained hemodynamically stable, and no repeat -butyl-2-cyanoacrylate injection was needed. He was finally discharged one week later and has been followed-up for the last one year with no signs and symptoms to suggest any recurrence of bleeding.

## Introduction

Ectopic varices have been reported to develop in various organs such as duodenum, colon, gall bladder, uterus, vagina, urinary bladder, and abdominal stomas [1-7]. Although the recognition of varices at unusual sites has long been described in the literature, standard diagnostic and therapeutic procedures have not yet been established for intestinal varices, which are rarely formed on other parts of the digestive tract than the gastroesophageal region [8]. Several promising treatment have been reported, but bleeding ectopic varices are regarded as potentially life-threatening [9]. We experienced ruptured duodenal varices which were successfully treated with N-butyl-2-cyanoacrylate (Histoacryl) injection.

## Case Report

**Figure 1:**
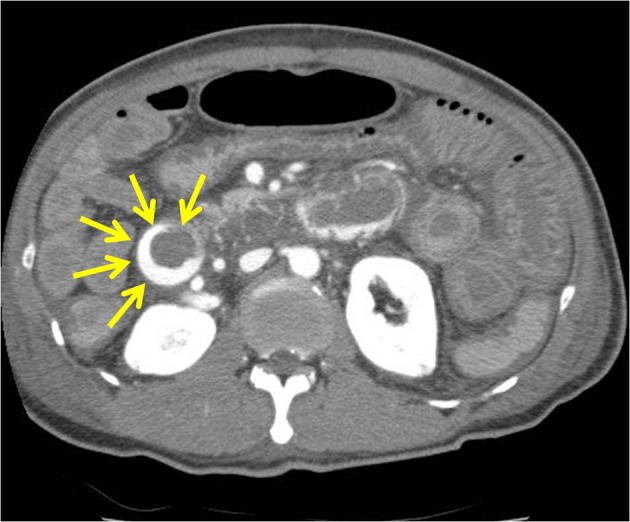
High attenuated lesion is observed at the third part of duodenum in the arterial phase of the abdominopelvic computed tomography scan (arrows).

**Figure 2:**
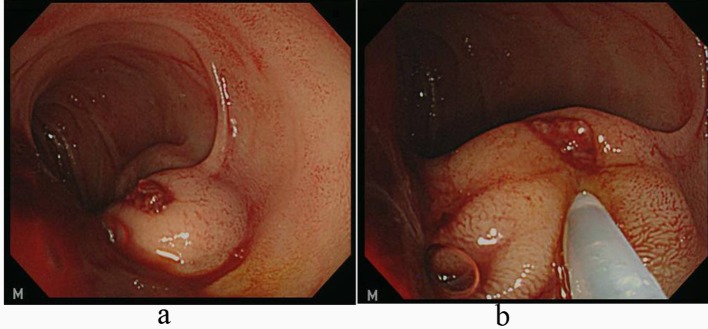
a: Emergent endoscopic findings of the distal third portion of duodenum using pediatric colonoscope demonstrate a blood-oozing erosion on the surface of large nodular duodenal varices; b: Mixture of N-butyl-2-cyanoacrylate and lipiodol is being injected.

**Figure 3:**
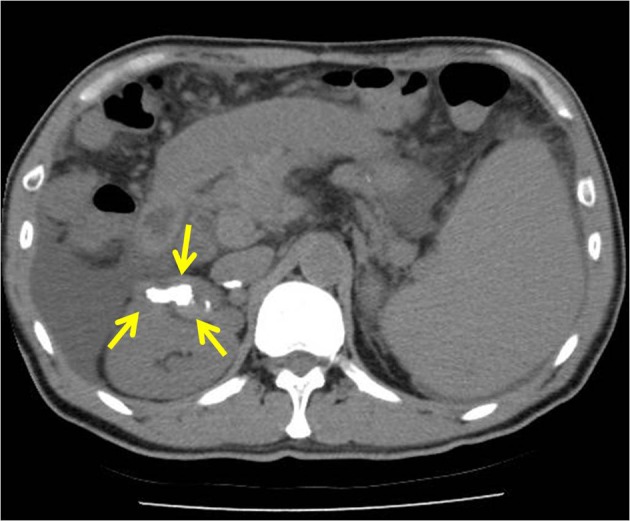
Computed tomography scan shows properly obliterated collateral vessels with N-butyl-2-cyanoacrylate and lipiodol (arrows).

A 65-year-old man had a known history of liver cirrhosis secondary to chronic hepatitis B infection. On this admission, he initially presented at the emergency department with melena for the past two days and hypotension. Hemoglobin level had dropped from 13.9 g/dL three weeks ago to 10.2 g/dL. At gastroscopy, no bleeding esophageal varices were seen, and no fresh blood or old blood clots were observed in the stomach and duodenum. However, active bleeding, shown in arterial phase, in the third part of duodenum was detected in following abdominopelvic CT examination (Fig. 1). Finally, a bleeding duodenal varix at the third portion, approximately 1.5 cm in diameter, was found by using pediatric colonoscopy (Fig. 2a). All 3 mL N-butyl-2-cyanoacrylate (histoacryl) was injected to the oozing duodenal varix, and active bleeding was successfully controlled (Fig. 2b). Subsequently, the patient remained hemodynamically stable, and no repeat N-butyl-2-cyanoacrylate injection was needed. Follow up CT, in pre-contrast phase, showed injected histoacryl eradicating the duodenal varix at the third portion of duodenum (Fig. 3). He was finally discharged one week later with a stable hemogloblin level of 12.5 g/dL. The patient has been followed-up for the last one year with no signs and symptoms to suggest any recurrence of bleeding.

## Discussion

Bleeding from ectopic varices is rare and accounts for only 1% and 5% of all variceal bleeding [1]. However, once the bleeding starts, it becomes difficult to control and is sometimes fatal. A surgical resection or interventional embolization of varices is sometimes useful when the varices are localized. However, surgical options, such as a portosystemic shunt or variceal ligation, are limited to selected patients. Unfortunately, many patients are not good operative candidates for such treatment modalities. The transjugular intrahepatic portosystemic shunt (TIPS) procedure is an effective modality in the therapy of cirrhotic patients with bleeding from ectopic varices unresponsive to conservative management [10, 11]. However, several data showed that TIPS frequently fail to eradicate gastric varices; the reported success rate is only 50% [12]. Moreover, there is recent evidence showing a higher re-bleeding rate after creation of a TIPS, compared to trans-catheter sclerotherapy [13]. Endoscopic procedures are relatively faster and easier than surgery or radiologic intervention. Among two major endoscopic management, endoscopic variceal ligation and endoscopic injection sclerotherapy, endoscopic variceal ligation is not entirely effective in blocking the flow of blood to the affected area and EVL can cause ulcers [14]. Therefore, endoscopic injection sclerotherapy seems to be favored for ruptured duodenal varices [15]. N-butyl-2-cyanoacrylate injection completely blocks a blood supply to a relatively large area and causes less tissue damage than other agents. In our case, N-butyl-2-cyanoacrylate with lipiodol injection was useful in achieving hemostasis.

In conclusion, we experienced a rare case of a rupture of ectopic varices located on the third portion of the duodenum. Endoscopic injection sclerotherapy with N-butyl-2-cyanoacrylate was proved to be very effective in treating ruptured duodenal varices.
